# Atlantic Salmon (*Salmo salar*) Cage-Site Distribution, Behavior, and Physiology During a Newfoundland Heat Wave

**DOI:** 10.3389/fphys.2021.719594

**Published:** 2021-08-24

**Authors:** Anthony K. Gamperl, Zoe A. Zrini, Rebeccah M. Sandrelli

**Affiliations:** Department of Ocean Sciences, Memorial University, St. John's, NL, Canada

**Keywords:** salmon, temperature, heart rate, electrocardiogram, activity, depth, heart rate variability, data storage tags

## Abstract

**Background:** Climate change is leading to increased water temperatures and reduced oxygen levels at sea-cage sites, and this is a challenge that the Atlantic salmon aquaculture industry must adapt to it if it needs to grow sustainably. However, to do this, the industry must better understand how sea-cage conditions influence the physiology and behavior of the fish.

**Method:** We fitted ~2.5 kg Atlantic salmon on the south coast of Newfoundland with Star-Oddi milli-HRT ACT and Milli-TD data loggers (data storage tags, DSTs) in the summer of 2019 that allowed us to simultaneously record the fish's 3D acceleration (i.e., activity/behavior), electrocardiograms (and thus, heart rate and heart rate variability), depth, and temperature from early July to mid-October.

**Results:** Over the course of the summer/fall, surface water temperatures went from ~10–12 to 18–19.5°C, and then fell to 8°C. The data provide valuable information on how cage-site conditions affected the salmon and their determining factors. For example, although the fish typically selected a temperature of 14–18°C when available (i.e., this is their preferred temperature in culture), and thus were found deeper in the cage as surface water temperatures peaked, they continued to use the full range of depths available during the warmest part of the summer. The depth occupied by the fish and heart rate were greater during the day, but the latter effect was not temperature-related. Finally, while the fish generally swam at 0.4–1.0 body lengths per second (25–60 cm s^−1^), their activity and the proportion of time spent using non-steady swimming (i.e., burst-and-coast swimming) increased when feeding was stopped at high temperatures.

**Conclusion:** Data storage tags that record multiple parameters are an effective tool to understand how cage-site conditions and management influence salmon (fish) behavior, physiology, and welfare in culture, and can even be used to provide fine-scale mapping of environmental conditions. The data collected here, and that in recent publications, strongly suggest that pathogen (biotic) challenges in combination with high temperatures, not high temperatures + moderate hypoxia (~70% air saturation) by themselves, are the biggest climate-related challenge facing the salmon aquaculture industry outside of Tasmania.

## Introduction

The marine environment is becoming warmer, and experiencing more frequent and severe heat waves and hypoxic episodes as a result of climate change (Breitburg et al., 2018; Frölicher et al., 2018; Oliver et al., 2018; Holbrook et al., 2019; IPCC, 2019; Sampaio et al., 2021). Further, these conditions, which can co-occur at marine sea-cage sites (Burt et al., 2012, Stehfest et al., 2017), may become a challenge for cage-cultured fish species and negatively affect growth, immunology, reproduction, and welfare (Reid et al., 2019). For example, it was recently reported that sea-caged Atlantic salmon (*Salmo salar*) in Tasmania were exposed to temperatures as high as 23°C and oxygen levels as low as 35% air saturation, and that these environmental conditions limited the depth distribution of the fish in the cages (i.e., led to crowding), and negatively affected feeding and filet quality (i.e., filet coloration) (Stehfest et al., 2017; Wade et al., 2019).

There are several studies that have examined the impact of summer environmental conditions on the distribution of fishes in sea-cages and the temperatures and oxygen levels that they prefer and/or avoid (e.g., Johansson et al., 2007, 2009; Oppedal et al., 2011; Stehfest et al., 2017). Further, researchers have recently used heart rate data storage tags (DSTs) to examine the effects of cage-site conditions and aquaculture practices (e.g., crowding, netting, brailing, and transportation) on aspects of trout/salmon physiology, stress, and welfare (Brijs et al., 2018, 2019; Hjelmstedt et al., 2020; Hvas et al., 2020; Føre et al., 2021; Svendsen et al., 2021). However, to date, no studies have combined these technologies to examine how summer cage-site conditions affect the depth distribution, behavior/activity, and heart rate of Atlantic salmon; the predominant finfish species reared in North America, Europe, Chile, and Tasmania, and whose annual global production was ~2.6 million tons in 2019 (worth ~ US $12.5 billion) (Food Agriculture Association of the United Nations, 2020). Such information is critical as heart function/rate is a key determinant of fish thermal tolerance and a good metric of stress and metabolic expenditures (Armstrong, 1986; Lucas, 1994; Wang and Overgaard, 2007; Anttila et al., 2014; Eliason and Anttila, 2017), and activity/movement makes up a large proportion of the energy budget of a fish (Clark et al., 2010; Gleiss et al., 2010). Further, understanding the spatial and temporal variability of key environmental variables within sea-cages, and how salmon respond to them, may enable modifications to farm siting and/or cage-site structure or management/operations that will improve fish health, welfare, and production/sustainability of the industry.

Recently, our research group performed lab-based tests to examine the feasibility and validity of using Star-Oddi (https://www.star-oddi.com; Gardabaer, Iceland) centi-HRT ACT DSTs (which measure temperature; electrocardiograms, ECGs; heart rate, f_H_; and heart rate variability, HRV; and 3-D acceleration), in combination with Milli-TD tags (which measure temperature and depth) on Atlantic salmon (Zrini and Gamperl, 2021). These studies showed that Star-Oddi's parameters of external acceleration (EA; a calibrated and normalized calculation of the vector of dynamic body acceleration, or VeDBA) and VAR (variation in EA) can be used to estimate swimming speed/activity and to discern certain swimming behaviors (i.e., burst-and coast swimming), respectively. Further, they showed that f_H_ is sensitive to changes in temperature, swimming speed, and diel rhythms, and that HRV may provide novel information on the cardiac function and physiology of free-swimming fishes.

Thus, to examine how present-day summer cage-site conditions influence the distribution, physiology, and activity of Atlantic salmon on the East Coast of Canada, we fitted 12 salmon with milli-HRT ACT and Milli-TD tags in mid-July 2019, released them into a sea-cage on the south coast of Newfoundland, and recovered them at harvest (in October). This produced a very comprehensive and unique dataset, in particular, because seawater temperatures were amongst the highest on record in Newfoundland in August (i.e., up to 19.5°C). The results of this study: add further support for using this type of technology for monitoring cage-site conditions and how it relates to fish physiology, behavior, and welfare; provide key information on how cage-site temperature impacts the physiology, distribution, and behavior of Saint John River origin salmon; and offer key insights into why lab-based studies of the thermal tolerance and immunology on these salmon (e.g., refer to Gamperl et al., 2020; Zanuzzo et al., 2020) would not have predicted the significant mortalities that occurred in these cages under these environmental conditions.

## Materials and Methods

### Location, Experimental Fish, and Research Statement

Twelve adult sea-caged Atlantic salmon (*Salmo salar*) with an average mass of 2.61 ± 0.15 kg (1.80–3.39 kg) and a fork length of 60.7 ± 0.97 cm (55.0–65.0 cm) were used in these experiments. The salmon were tagged in a single sea-cage at a site located on the south coast of Newfoundland (Canada). The fish were grown under typical production protocols in a circular sea-cage (100 m in diameter and 15 m deep with an extended conical bottom) stocked with ~35,000 fish. This research was conducted with the permission and assistance of the aquaculture company with which this work was conducted. However, based on a non-disclosure agreement, we cannot provide information on the exact location of the cage-site, the environmental data they collected, or the number of mortalities that occurred in the sea-cage in which the fish were placed.

### Data Storage Tags Implantation and Attachment

Salmon were simultaneously implanted/equipped with two DSTs produced by Star-Oddi. The Milli-TD tag (diameter, 13 mm; length, 39.4 mm; weight in air 12 g; version 15 DM/CRC16/4800) records depth (0–100 m; 0.6% accuracy) and temperature (−3 to 40°C; ± 0.1°C accuracy). The milli-HRT ACT tag (diameter, 13 mm; length, 42 mm; weight in air 12 g; version 35 CRC16/4800/MSO/RST) records heart rate, tri-axial acceleration (with a resolution of ± 2 mg), and temperature (5–45°C; accuracy up to ± 0.2°C). The time recorded from both tags is accurate to ± 1 min month^−1^. The combined mass of the tags did not exceed 2% of the body mass of the fish, and thus, was consistent with the recommendations/findings for the use of tags in fish (Makiguchi and Kojima, 2017; Wright et al., 2019).

Prior to implantation, the DSTs were inserted into Star-Oddi's tag-computer interface (COM-BOX). Both tags were set (using Sea Star software for the Milli-TD tags and Mercury software for the milli HRT-ACT tags) to begin recording on July 11th, 2019 at 4:00 a.m. Newfoundland Standard Time (UTC-3:30). The Milli-TD tags were set to record depth and temperature every 5 min. The milli-HRT ACT tags were set to record heart rate (based on ECGs recorded at 100 Hz for 15 s), acceleration (at 1 Hz for 60 s), and temperature every 2 h, and to save the associated electrocardiograms (ECGs) and raw accelerometry data every fourth measurement. The Milli-TD tags were prepared for external attachment using Star-Oddi's plate holder kit, which consisted of two silicone pads and two plastic molds. Prior to implantation, two flexible stainless steel wires (0.02” diameter) were looped around the tag and the ends were passed through the larger silicone pad and the pre-drilled holes of the larger plastic mold. The milli-HRT ACT tags were prepared for internal implantation by tying two 30 cm pieces of black, braided, non-absorbable, and non-sterile silk suture (2-0) threads around the tag (see Zrini and Gamperl, 2021).

On July 10th, 2019, the salmon were netted from the sea-cage into a live well aboard a boat stationed next to the cage. One at a time, salmon were netted from the live well into a tote containing 0.2 g L^−1^ tricaine methanesulfonate (MS-222). The fish were then implanted/fitted with the tags following the surgical methods described in Zrini and Gamperl (2021). Briefly, when the fish reached a surgical plane of anesthesia (loss of equilibrium and unresponsive to stimuli), it was placed ventral side upon a wetted sponge on a surgical table, and the gills were continuously irrigated with seawater containing 0.1 g L^−1^ MS-222. A ~3 cm, mid-ventral, incision was made posterior to the location of the pericardium using a scalpel. The milli-HRT ACT logger was then inserted posteriorly toward the tail of the fish with the two parallel ECG electrodes facing the muscle tissue and then pulled forward toward the pericardium. The sutures attached to the tag were then tied to the body wall of the salmon at the anterior and posterior margins of the incision. Finally, the incision was closed with continuous stitches using 3-0 sterile silk suture.

The fish were then turned over, and four stainless steel hypodermic needles (15 gauge, 3.5” long) were passed through the skin and muscle below the dorsal fin. The wires attached to the tag and plate holder kit were guided through the needles, the needles were removed, and the four wires exiting the muscle were passed through a silicone pad and plastic mold, and twisted together [refer to Zrini and Gamperl (2021) for pictures]. Fish were then returned to a ventral side up position, and Vet-bond and Polysporin® were applied to the incision. The surgery took an average of 12 ± 2 min (range 10–17 min). Prior to being returned to the sea-cage, the salmon were given time to recover in the live well until they were actively swimming (~10–15 min).

The fish and tags were recovered for one of two reasons: they were a pre-harvest mortality or at harvest. Fish that died in the cage sunk to the bottom, and were collected by divers of the company. Between October 3rd and 7th, the fish were harvested, and the tags were removed by workers on the processing line of the company. The fish were identified by workers using the external Milli-TD tags. No post-mortem dissections or measurements were conducted in this experiment. After retrieving the tags from the company, the data was downloaded using the COM-BOX and Mercury/SeaStar software (Star-Oddi).

### Data Analyses

All measurements of heart rate (*f*_H_) were provided with a unit-less measurement known as the quality index (QI) determined by the Mercury software. This measurement represents the quality of the ECG signal, where QI_0_ means very good quality and indicates that the variation in the R-R interval was <20% during the 15 s recording, QI_1_ and QI_2_ indicate decreasing quality, and QI_3_ indicates that no R-R interval was detected (i.e., the R peak in the PQRS complexes could not be clearly distinguished or two R peaks were not visible in the ECG) (Star-Oddi). Manual calculations of *f*_H_ were performed on all stored ECGs (251 per fish). To calculate *f*_H_, the time between R wave peaks was measured in seconds and averaged, and then 60 was divided by the average to obtain *f*_H_ in beats per minute (bpm). Manual calculations were not possible for most QI_3_ categorized values or when ECG artifacts made the QRS complex unidentifiable and these data were removed. The absolute difference in *f*_H_ was calculated between the manual calculations of *f*_H_ and the value calculated by Star-Oddi's on board algorithm. Values of *f*_H_ <10 bpm or >150 bpm (max. *f*_H_ of 12°C-acclimated Atlantic salmon; Anttila et al., 2014) were also removed. HRV was calculated from the stored ECGs as the SD of the time between successive R wave peaks (in ms).

The milli-HRT ACT tag records when the sensor is measuring acceleration above standard gravity and the software calculates EA as a vectorial sum dynamic of body acceleration, or VeDBA, (measured in m-g at 1 Hz for 60 s, where g is the acceleration of gravity or 9.8 m s^−2^), which is then averaged over 1 min. Additionally, the software performs a 360-degree static calibration on each logger for each of the axes. The Mercury software reports minimum, maximum, and average EA values recorded over the 1 min sampling period, however, the raw data is also available if stored. VAR is the variance in EA calculated as the standard deviation squared over a set sampling period (measured in m-g^2^ at 1 Hz for 60 s). Values of VAR >222 m-g^2^ were used to determine when the fish was not constantly swimming; that is, they were engaged in burst-coast swimming (Zrini and Gamperl, 2021) or potentially struggling etc. Visual analysis of the raw activity data showed evidence that tag MAL0011 rotated ~90 degrees inside the fish and average and minimum EA values were higher than the other four fish or previously reported values (Zrini and Gamperl, 2021). A similar issue occurred with tag MAL0025 later in the trial as minimum EA values began to deviate from 0 m-g indicating movement of the tag unrelated to the movement of the fish, however, there was no evidence of the tag rotating. All EA and VAR data from tag MAL0011 and data after August 18th from tag MAL0025 were removed from the analysis. These issues did not affect the quality of the ECGs recorded in these tags.

The *f*_H_ and depth data were used to determine the exact time of death and removal from the sea-cage, respectively, of each harvested fish (Table 1). The earliest time of death was October 3rd at 4:00 a.m., and thus, data were analyzed between July 11th at 4:00 a.m. and October 3rd at 11:55 p.m. (i.e., over 84 days). Information on the sunrise and sunset on the south coast of Newfoundland between these times were retrieved from https://sunrise-sunset.org. Data were sorted into “day” and “night” values based on the time of sunrise or sunset for a given day. Day and night-time averages were calculated for *f*_H_, average EA, the percentage of values indicating non-steady swimming, the percentage of QI_0_ ECGs, depth, and temperature.

**Table 1:** Body measurements, the date of the last viable heart rate determined from stored ECGs, and the estimated time of harvest from the sea-cage determined from by the depth data.

**Fish number**	**Milli-HRT ACT**	**Milli-TD**	**Initial weight with tags (kg)**	**Length (cm)**	**Last reliable heart rate**	**Time of removal from the cage**
1	MAL0011	B2672	2.00	56	Oct. 7 12:00 AM	Oct. 7 6:45 AM
2	MAL0014	B2657	2.87	62	Oct. 3 12:00 AM	Oct. 3 11:50 AM
3	MAL0017	B2651	2.35	60	Oct. 7 12:00 AM	Oct. 7 6:50 AM
4	MAL0018	NA	3.35	65	Oct. 3 12:00 AM	NA
5	MAL0025	B2671	3.39	65	Oct. 7 12:00 AM	Oct. 7 10:15 AM
6	NA	B2669	2.15	58	NA	Oct. 4 10:00 AM
7	NA	B2653	1.80	55	NA	Oct. 4 12:20 PM
8	NA	B2666	2.84	61	NA	Oct. 3 11:55 AM

*NA represents tags that were not retrieved upon harvest*.

### Statistics

All graphing and statistical analyses were performed using Prism 8 (GraphPad Software, Inc., San Diego, CA, USA). Linear regression analyses were used to examine how the daily percentages of QI_0_, QI_1_, QI_2_, and QI_3_ ECGs, and the absolute difference in heart rate, changed over time. Average values of depth, temperature, heart rate, HRV, external acceleration, and percentage of non-steady swimming were calculated for 1-week periods (i.e., July 20–27, August 3–10, 17–24, August 27–September 3, and September 21–28). Two-way repeated measures ANOVAs were used to analyze this data with time and photoperiod as fixed-effects, followed by Bonferroni multiple comparison tests to determine statistical differences within photoperiod groups. Individual and average values of *f*_H_ and EA were plotted against their associated temperature values, and heart rate was plotted vs. external acceleration. The first 14 days of data were removed in this analysis to account for surgical recovery (Zrini and Gamperl, 2021). Linear regression analyses were used to examine the relationships between these parameters. The level of statistical significance used in all analyses was *P* < 0.05, and data in the figures, tables, and text are means ± SE.

## Results

### Fish Survival, Tag Recovery, and Harvest

Within the first 19 days following implantation, three tagged fish died; one fish was retrieved on July 12th, and two were retrieved on July 30th. In two of these fish, it appears that the surgical site had not completely healed based on limited notes taken by the divers. The data for the other fish were examined, and no deviations from that of the surviving fish were noted (i.e., *f*_H_, EA and depth values appeared normal/typical, and the former closely tracked changes in temperature (data not shown)). From these three fish, all six tags were recovered. Tags from eight out of nine of the remaining fish were retrieved (five milli-HRT ACT and seven Milli-TD tags). The reason(s) for the one missing fish is/are not known, however, it is possible that this fish died in the sea-cage and was not recovered by the divers. It is also possible that its tags were not collected or missed on the processing line. The initial weights and lengths, approximate time of death, and time of removal of the fish from the sea-cage are shown in Table 1.

### Heart Rate Calculations and Quality Analysis

The *f*_H_ measurements recorded by the tag had the following distribution of QI values during the experimental period: QI_0_ = 42.5 ± 7.3%, QI_1_ = 48.4 ± 6.7%, QI_2_ = 4.5 ± 2.1%, and QI_3_ = 4.6 ± 2.2%. Out of 1,007 *f*_H_ measurements recorded per fish, 251 associated ECGs were saved to the memory of the tag. From the saved ECGs, only 2.4 ± 2.1% of *f*_H_ values could not be calculated because of artifacts in the ECG rendering the PQRS complex unidentifiable. The measurements of *f*_H_ with saved ECGs were replaced with their manually calculated values. Of the newly adjusted and remaining original *f*_H_ values, 3.85 ± 1.8% were outliers (< 10 bpm or > 150 bpm) and were removed. The majority of the outliers were ≤ 2 bpm (~ 91%) indicating that the PQRS amplitude was misidentified by the on-board algorithm.

The quality of the ECGs decreased over the duration of the tagging trial as indicated by a decreasing percentage of QI_0_, or “good” quality, ECGs (y = −0.26x + 51.67; R^2^ = 0.30; *P* < 0.0001; data not presented). While the percentage of QI_1_ ECGs did not change (*P* = 0.9185), the percentage of ECGs with QI_2_ (y = 0.11x + 0.09; *R*^2^ = 0.36; *P* < 0.001) and QI_3_ values increased (y = 0.16x – 2.02; *R*^2^ = 0.58; *P* < 0.001). Further, the absolute difference between the values manually calculated and those calculated by the on-board algorithm increased over time (y = 0.10x – 0.22; *R*^2^ = 0.15; *P* < 0.001; data not presented). Nonetheless, the mean absolute difference in *f*_H_ was only 4.1 ± 0.40 bpm (ranging from 0 to 276 bpm), with 66.7% of *f*_H_ values being < 1 bpm different and 89.7% being < 5 bpm different. The absolute difference in *f*_H_ increased with QI value: QI_0_ = 1.0 ± 0.3 bpm, QI_1_ = 1.8 ± 0.7 bpm, QI_2_ = 19.6 ± 7.4 bpm, and QI_3_ = 45.8 ± 0.8 bpm.

### Cage-Site Conditions and Fish Distribution

**Figure 1:**
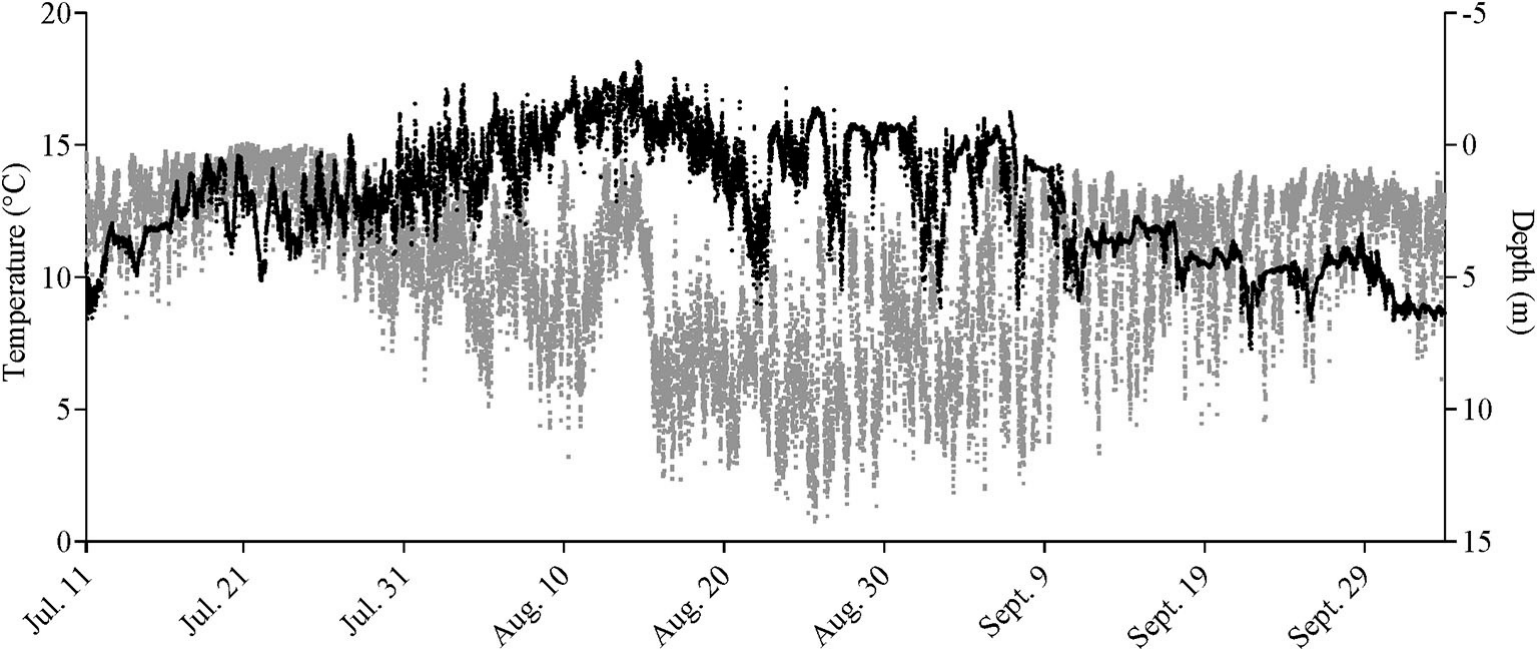
Mean depth (in gray) and temperature (in black) measured every 5min in seven Atlantic salmon fitted with Milli-TD tags over the duration of the study. For data on individual fish, see Supplementary Figure 1.

**Figure 2:**
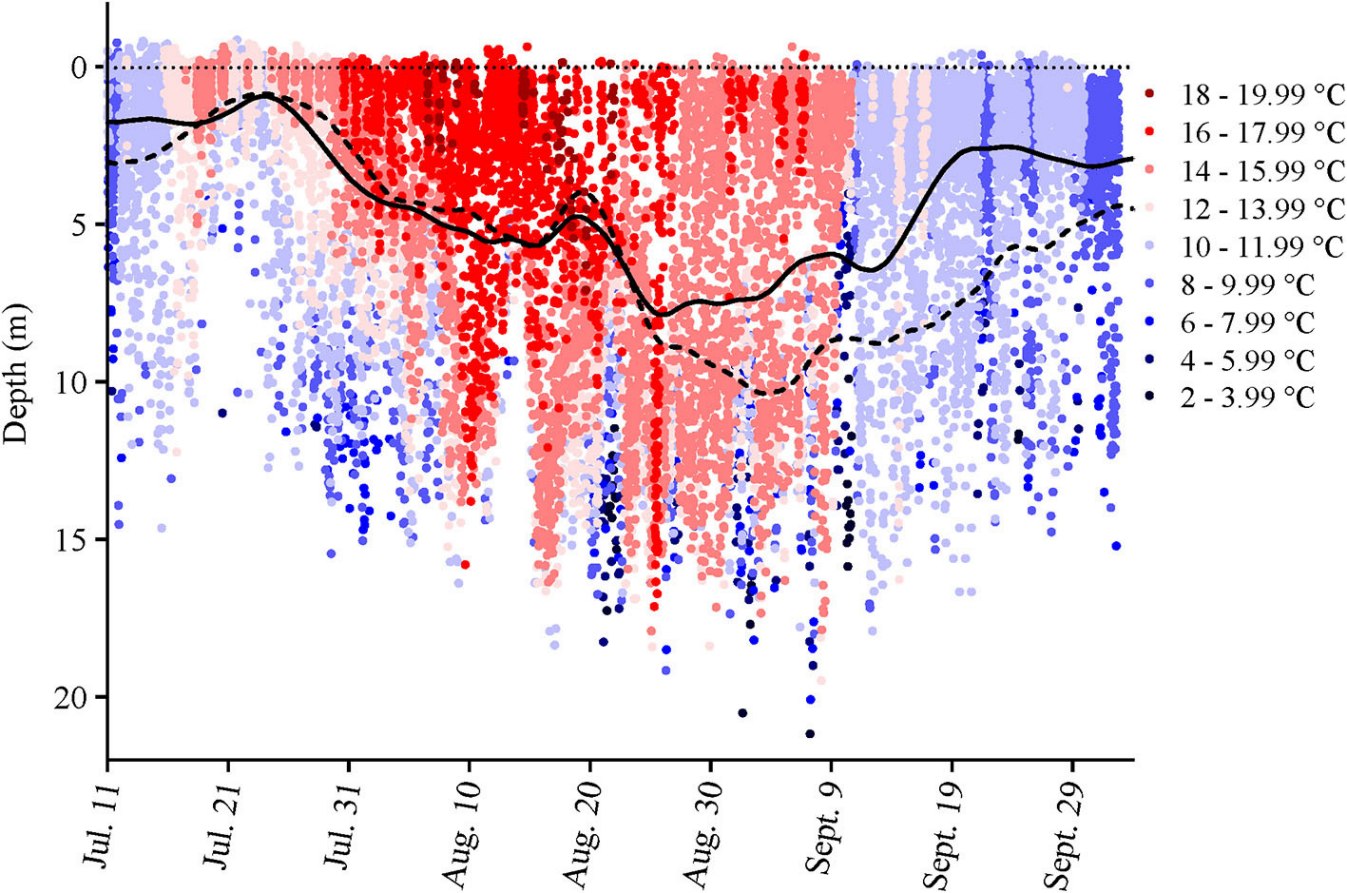
Cage-site temperature data at various depths over the duration of the study. The data were obtained from the Milli-TD tags that were attached to the seven salmon that were retrieved at the end of the study. The Lowess curves show the mean depth that the fish occupied during the day (broken line) and night (solid line).

Only a partial dataset was available for the water-oxygen level in this cage, and temperature data were only taken inside the cage perimeter at the surface and at 5, 10, and 15 m. Given the coarseness of this data, the fact that the tagged salmon were found at various depths over the course of a day (e.g., as shown in Figure 1, Supplementary Figure 1), and that the Milli-TD tag recorded depth and temperature every 5 min, we used the latter data to produce a detailed depth/temperature profile for the cage from July 11th to October 3rd (Figure 2).

**Figure 3:**
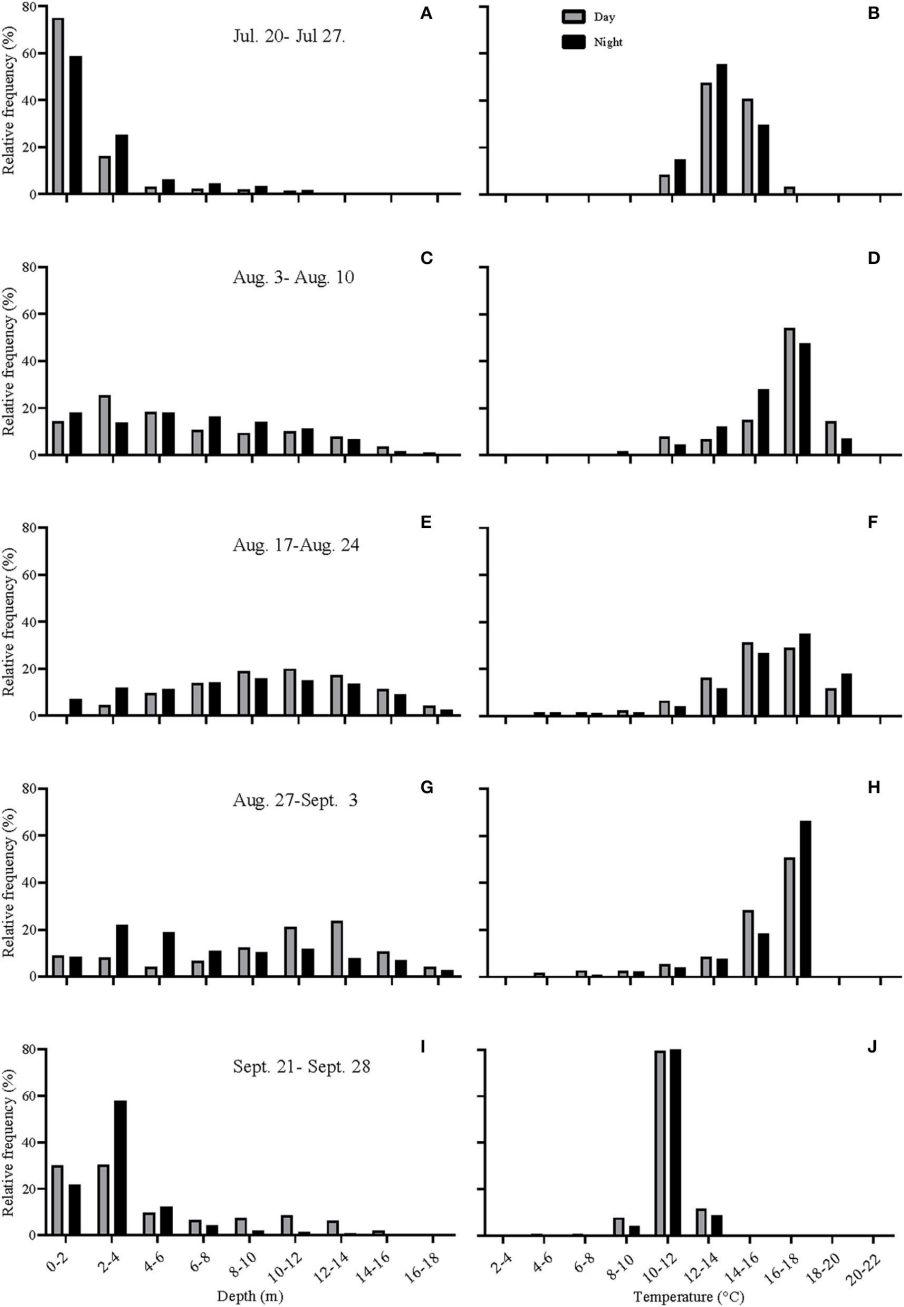
**(A–J)** Histograms showing the depth distribution and the temperatures experienced by the fish during various periods of the study. July 20th−27th, ~ 2 weeks after tag implantation; August 3rd−10th, period of highest surface water temperatures; August 17th−24th, start of mortalities in cage-sites in the region; August 27th–September 3rd; period of high mortalities in cage-sites in the region; September 21st−28th, after temperatures cooled down. Gray bars represent day-time values, whereas black bars represent night-time values.

During the first 5 days of recovery, temperatures in the cage were relatively homogenous, with temperatures ranging from 10 to 12°C, with the fish mainly occupying the top 2–3 m (Figures 1–3; Supplementary Figure 1). After this point, the surface waters (< 2 m) warmed to 14–16°C, and the fish frequented these depths despite having access to cooler (8–10°C) waters deeper in the cage (Figures 1, 3A,B). Beginning around July 30th, there was a gradual increase in surface water temperatures and the depth that warm temperatures reached. For example, water temperatures were generally 16–18°C (but occasionally above 18°C) at depths down to 5 m, and the depth at which temperatures were above 14°C gradually increased from ~2–3 to 15 m on August 16th (Figures 1, 3C,D). During this period, the mean depth of the fish gradually increased to ~5 m, although the fish went as deep as 12–15 m and showed little depth preference (Figures 1, 3C). Temperatures at the surface of the cage remained >16°C over the next week (~ August 16th−22nd), and the salmon made limited use of surface waters (Figures 1, 2, 3E). During this week, maximum temperatures were recorded for four of the seven fish (range 19.2–19.4°C) (Table 2), with the average 10 highest temperatures for all fish ranging from 18.5 to 19.1°C (Table 3). Thereafter, the fish moved slightly deeper in the cage, and the mean day-time and night-time depths between August 22nd and September 9th were ~ 9–10 and 6–7 m, respectively. However, they also spent a considerable amount of their time (~40%) below 10 m during this period, and some fish were occasionally measured at depths between 15 and 18 m (the latter likely due to the conical nature of the bottom of the cage, and possibly the accumulation of mortalities) (Figures 1, 3G). They remained at these depths (where water temperatures were ~14–16°C) until water temperatures became considerably cooler about September 9th when hurricane Dorian passed over Newfoundland and this resulted in a mixing of the water column (i.e., homogenous water temperatures with depth; ~8–12°C). After this change in temperature, the mean depth that the fish occupied gradually decreased, but again the mean depth of the salmon during the day was ~2 m deeper than that observed at night; these values ~3 and 5 m, respectively (Figures 2, 3I). This was because, although diurnal patterns of depth preference were often observed over the course of the study for all fish to this point, diurnal movement (depth) patterns (shallower during the night, deeper during daylight hours) were particularly evident for many fish during this final period (as shown in the Supplementary Figure 1).

**Table 2:** The highest temperature experienced by each salmon based on the data from their external tags (Milli-TD, fish numbers listed on top of each column), and the date, depth, and time of day that this value was recorded.

	**Fish number**						
	**1**	**2**	**3**	**5**	**6**	**7**	**8**
Date	Aug. 21	Aug. 7	Aug. 7	Aug. 21	Aug. 7	Aug. 21	Aug. 21
Time	9:25 PM	4:55 PM	10:40 PM	9:30 PM	4:55 PM	8:55 PM	9:30/
Temp.	19.41°C	19.37°C	19.12°C	19.18°C	19.49°C	19.42°C	19.28°C
Depth	0.58 m	0.00 m	0.33 m	3.33 m	0.56 m	0.12 m	1.77

**Table 3:** The average 10 highest temperatures (°C) per time period, recorded from the external tags (Milli-TD, fish numbers listed on top of each column) that were attached to the salmon.

	**Fish number**						
	**1**	**2**	**3**	**5**	**6**	**7**	**8**
July 20–27	15.3 ± 0.03	15.4 ± 0.04	15.5 ± 0.06	15.5 ± 0.06	15.5 ± 0.04	15.2 ± 0.03	15.2 ± 0.03
Aug. 3–10	19.0 ± 0.18	18.8 ± 0.42	18.9 ± 0.12	18.4 ± 0.16	19.2 ± 0.14	17.7 ± 0.41	18.6 ± 0.20
Aug. 17–24	19.1 ± 0.19	18.8 ± 0.12	18.5 ± 0.05	18.7 ± 0.27	18.7 ± 0.36	19.0 ± 0.36	19.1 ± 0.15
Aug. 27–Sept. 3	16.2 ± 0.06	16.2 ± 0.08	16.1 ± 0.07	16.1 ± 0.09	16.2 ± 0.06	16.2 ± 0.06	16.4 ± 0.03
Sept. 21–28	11.8 ± 0.06	11.8 ± 0.03	11.4 ± 0.08	11.9 ± 0.08	12.1 ± 0.08	11.6 ± 0.03	11.7 ± 0.06
July 11–Oct. 3	19.2 ± 0.17	19.0 ± 0.19	18.9 ± 0.12	18.7 ± 0.25	19.3 ± 0.13	19.0 ± 0.32	19.1 ± 0.15

*Data are means ± S.E*.

### Heart Rate and Activity

**Figure 4:**
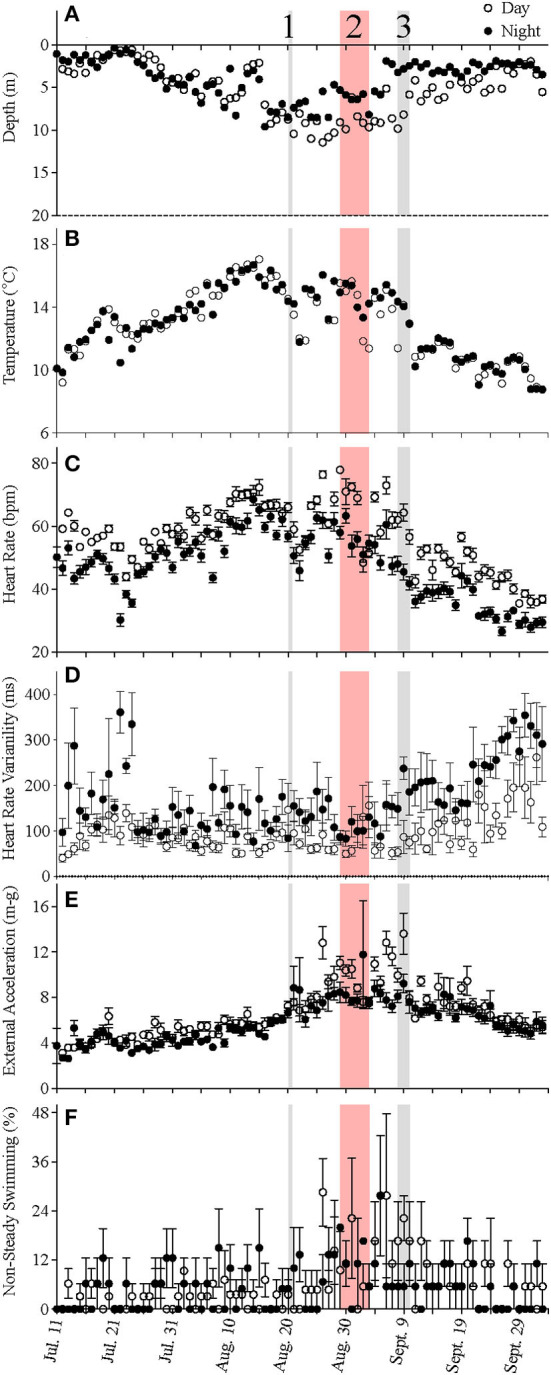
**(A–F)** Average day-time (open circles) and night-time (filled circles) values for depth, temperature, heart rate, heart rate variability, external acceleration, and the percentage of non-steady swimming measured for five fish over the duration of the study. Values are means ± S.E. (*N* = 5). 1—indicates the day that feeding was stopped or greatly reduced until surface water temperatures fell below 18°C; 2—indicates the period when there were mass mortalities in many cage-sites in the region where this cage-site was located. 3—indicates when hurricane Dorian moved over the south coast of Newfoundland.

**Figure 5:**
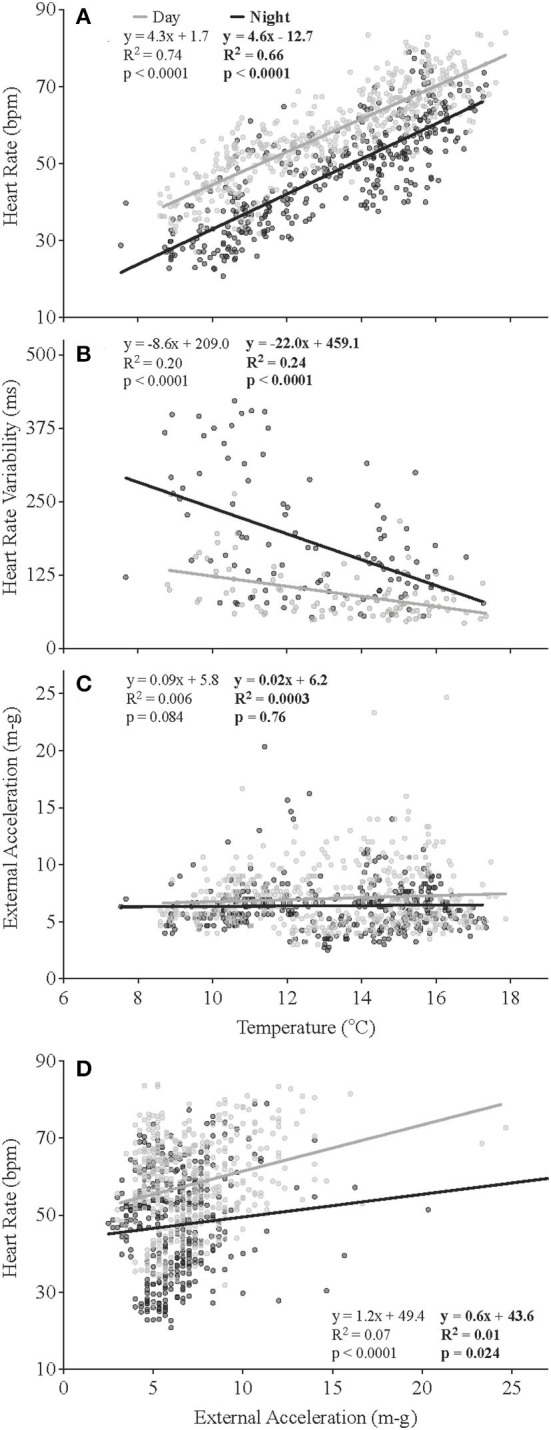
Relationships between recorded temperatures and heart rate **(A)**, heart rate variability **(B)** and external acceleration **(C)**, and between heart rate and external acceleration **(D)**. Each plot represents 838 individual data points (**B**, 209 data points calculated from saved ECGs) recorded over the duration of the study and excludes the first 14 days of data to account for surgical recovery (*N* = 5).

At the start of the experiment, when mean temperature experienced by the fish was ~10°C, heart rates were ~60 bpm during the day and 50 bpm during the night (Figure 4C). The salmon's mean f_H_ fell to ~47 bpm after 2 weeks of recovery, despite water temperature being slightly warmer (~12–14°C). This higher heart rate during the day, based on the data for individual fish, was normally observed for the duration of the experiment. Changes in mean heart rate closely followed those of the temperature of the fish (Figure 5, see Supplementary Figure 2 for individual data), with a heart rate during the peak temperature period (mean ~ 17°C) on August 15th of ~70–80 bpm (Figure 4C). Thereafter, heart rates generally decreased and reached ~ 37 and 28 bpm, respectively, prior to harvest when mean water temperatures were ~6–8°C. Elevated values of HRV were recorded in the night-time for the first 17 days following surgery, and average day-time and night-time values were ~109 and 189 ms, respectively, between July 20 and 27th (Table 4, Figure 4D). HRV, while ~40 ms higher in the night-time, remained low during the trial until temperatures began to decrease following hurricane Dorian. Heart rate variability was significantly related to temperature (Figure 5B) and night-time values reached a peak of 270.8 ± 10.9 ms when temperatures were 10.2°C. External acceleration averaged ~4 m-g at the beginning of the experiment, increased to ~8 m-g between August 25th and September 9th, and then decreased gradually (Figure 4E). In the above timeframe, values of EA during the day were much higher than during the night (by ~ 4 m-g), and this increased activity was associated with a much greater percentage of activity (EA) measurements that could be categorized as non-steady swimming (~ < 10 vs. >20%, Table 4, Figures 2D,E). Further, although there was no overall relationship between EA and heart rate (Figure 5C), this period was associated with the biggest difference in heart rate during the day vs. the night (Figure 4C).

**Table 4:** Average day-time and night-time (gray shading) values for depth, temperature, heart rate, and measures of activity for the sea-caged salmon during particular 1-week–periods: i.e., July 20th−27th, ~ 2 weeks after tag implantation; August 3rd−10th, period of highest surface water temperatures; August 17th−24th, start of mortalities in cage-sites in the region; August 27th–September 3rd; period of very mortalities in cage-sites in the region; September 21st−28th, after temperatures cooled down.

	**Depth (m)**	**Temp. (°C)**	**Heart rate (bpm)**	**Heart rate variability (ms)**	**External acceleration (m-g)**	**Non-steady swimming (%)**
July 20–27	1.1 ± 0.2^a^	12.7 ± 0.1^a^	51.7 ± 1.9^a^	109.1 ± 21.4	4.5 ± 0.3^a^	2.0 ± 0.7
	1.8 ± 0.4^A^	12.3 ± 0.1^A^	41.9 ± 1.8^A^	189.1 ± 15.4^A^	3.7 ± 0.2^A^	1.6 ± 0.9
Aug. 3–10	5.2 ± 0.7^b, c^	15.1 ± 0.4^b^	62.9 ± 2.0^b^	76.7 ± 5.4	5.3 ± 0.5^a, b^	3.3 ± 1.8
	5.7 ± 0.7^B^	14.9 ± 0.3^B^	55.1 ± 2.1^B^	129.9 ± 9.4^AB^	4.6 ± 0.4^A, B^	4.7 ± 1.9
Aug. 17–24	9.1 ± 0.6^d^	14.0 ± 0.4^a, b^	62.2 ± 2.0^b^	91.2 ± 13.7	7.3 ± 0.8^a, b^	2.7 ± 1.2
	7.6 ± 0.7^B^	14.5 ± 0.5^B^	55.7 ± 1.6^B^	140.2 ± 16.7^A, B^	7.3 ± 0.9^A, B^	3.8 ± 2.4
Aug. 27–Sept. 3	8.9 ± 1.1^b, d^	14.0 ± 0.4^a, b^	66.4 ± 0.8^b^	84.1 ± 18.1	10.1 ± 0.6^b^	10.9 ± 2.4
	6.0 ± 0.9^BC^	14.7 ± 0.3^B^	56.4 ± 1.0^B^	107.3 ± 15.7^B^	9.1 ± 0.7^B^	9.9 ± 2.2
Sept. 21–28	3.9 ± 1.0^a, c^	10.1 ± 0.1^c^	42.9 ± 1.2^c^	157.5 ± 35.1	8.1 ± 2.0^a, b^	4.2 ± 1.5
	2.3 ± 0.3^A, C^	10.2 ± 0.0^C^	30.8 ± 0.6^C^	270.8 ± 10.9^C^	7.8 ± 2.2^A, B^	1.6 ± 1.0

*Values are means ± S.E. Within a column, values without a letter in common are significantly different (P < 0.05), N = 5. Note: data for the day and night were analyzed separately. Dissimilar lower and upper case letters indicate day-time and night-time values, respectively, that are significantly different*.

## Discussion

### Fish Survival and Tag Recovery

We attribute the early mortality of the three fish to complications related to surgery (e.g., poor wound healing), and/or the combined stressors of surgical procedures and anesthesia (Wright et al., 2019; Hvas et al., 2020). The experience level of the surgeon has been shown to significantly alter wound healing and recovery of rainbow trout (*Oncorhynchus mykiss*; Hjelmstedt et al., 2020). However, this was unlikely to have been the cause of the salmon mortalities in this study as the surgeon (Z.A.Z.) had considerable experience (see Zrini and Gamperl, 2021; Zrini et al., 2021) and surgical times were short (average 12 min). That said, recovery location might have been a contributing factor. For example, Brijs et al. (2018) were able to recover rainbow trout in a facility for 1 week prior to releasing them back into the sea-cage and had 0% mortality. A recovery facility was not available for this project but may increase survival in future studies. The missing tags may have been expelled/become unattached in the cage, not been identified on the processing line, or could have been associated with mortalities that were not recovered from the cage. The latter explanation is certainly possible as Macaulay et al. (2021) indicate that the percentage of tagged fish that die increases markedly with the duration of the study/trial, and our study lasted >2.5 months.

### Salmon Distribution and Behavior

#### Salmon Depth Distribution

For the first week to 10 days post-implantation, the salmon remained in the top 0–4 m of the cage and made infrequent excursions into waters deeper than 10 m. However, as surface temperatures increased, their mean depth increased to 8–10 m, and they made frequent trips to deeper and cooler waters (Figures 1, 3, 4, Supplementary Figure 1). While their depth was influenced by a number of factors (see below), it is apparent that when access to a variety of water temperatures was available, they preferred temperatures from ~14 to 18°C, and generally avoided temperatures > 18°C. This data fits with the notion that temperature is the primary (most influential) factor affecting salmon depth distribution in sea-cages (Oppedal et al., 2011) and with the preferred temperatures of 13.5°C (Sutterlin and Stevens, 1992) and 16.5–17.5°C (Johansson et al., 2006) reported for Atlantic salmon in net pens. Further, this data is consistent with temperatures reported for the optimum growth of this species (14–18°C: Jobling, 1981; Sambraus et al., 2018). When temperatures were at peak levels (~ July 30th–August 25th), the mean depth of the fish increased (see above). However, it was clear that most of the fish continued to utilize the entire depth of the cage (i.e., they were not restricted to, or “crowding” at, particular depths), and that there were no abrupt temperature changes with depth (Figures 1, 2, Supplementary Figure 1).

For the first 5–6 weeks, there was little difference in the mean depth occupied by the salmon during the day vs. the night (although this pattern was still discernable for individuals). This may have been partially related to the fact that temperatures above 12°C were only present over a very narrow range of depths (i.e., generally in the top 2–3 m; Figure 2). Indeed, earlier studies suggest that the temperature preference of salmon is more strongly expressed when temperature stratification is more prominent (Oppedal et al., 2007). In contrast, after ~ August 25th, a clear diurnal pattern in mean depth occurred with fish ~2–4 m deeper during the day than night. This diurnal pattern of depth distribution has been reported by several authors for salmon (refer to Oppedal et al., 2011), and may have been enhanced after August 20th due to the cessation of feeding and/or reduced feeding. Nonetheless, we did not observe that the salmon utilized more of the cage depth at night (Figure 3) as has been reported by previous authors (Oppedal et al., 2001; Dempster et al., 2008; Korsøen et al., 2009).

#### Swimming Activity

External acceleration generally ranged from 4 to 8 m-g over the study and the percentage of time that they were estimated to be engaged in non-steady (i.e., burst-and-coast) swimming was generally below 10–12% (Figures 4E,F). Based on the swim tunnel calibrations performed by Zrini and Gamperl (2021), these values of EA equate to swimming speeds from 0.4 to 1.0 body lengths per second (~ 25–60 m s^−1^). This range of values is very close to that reported by other authors for adult (large) salmon when using underwater cameras (Korsøen et al., 2009; Oppedal et al., 2011). There were noticeable differences in EA and the percentage of time spent non-steady swimming over the course of the study. EA was ~4 m-g at the start of the study, rose steadily to ~8 m-g by August 25th, remained at this level for ~2 weeks, and then began to decline. Swimming speed has been reported to vary with season (Oppedal et al., 2001), and the observed pattern in EA/swimming speed would appear to be related to temperature based on the similar pattern of change in these two parameters (i.e., compare Figures 4B,E) over the course of the study. However, there was absolutely no relationship between temperature and EA based on analysis of the mean data (Figure 5C) or of individual fish (Supplementary Figure 2). Sea-cages are known to have environmental conditions that are highly variable with space and time (Oppedal et al., 2011), and other factors such as day length, water currents, etc., must have influenced fish locomotor activity. Interestingly, EA and the percentage of values indicating non-steady swimming were highest between ~ August 25th and September 8th, particularly during the day. This increase in EA and the percentage of non-steady swimming may have been related to the cessation of, or reductions in, feeding at the cage-site when surface temperatures exceeded 18°C (Figure 3). When hungry, fish may increase swimming speed to increase their chance of finding food (Kadri et al., 1991; Boisclair, 1992). However, the influence of long-term reductions in feeding, or food deprivation, on fish activity/swimming speed, and behavior has yet to be reported.

### Heart Rate and Heart Rate Variability (HRV)

#### Heart Rate Calculations and Quality Analysis

The quality of ECGs recorded decreased over the duration of the tagging trial and was associated with an increase in QI_2_ and QI_3_ values. QI_2_ values indicate that the ECGs had reduced quality and QI_3_ values indicate that no R-R intervals were identified. Reductions in ECG quality could be due to the movement of the tag during the trial; however, this cannot be substantiated as post-mortem dissections were not conducted. While there was evidence of tag MAL0011 rotating inside Fish 1, this was only associated with changes in the activity measurements and not decreases in ECG quality. It is possible that the sutures loosened in the other fish and that the tag moved to a position slightly further from the heart, resulting in reduced quality of the ECGs (Brijs et al., 2018). Nonetheless, QI_0_ and QI_1_ were still < 2 bpm from manually calculated values, and thus, this was not a major issue with regard to the accuracy of the collected data. The increased number of QI_3_ ECGs was associated with low *f*_H_ values later in the summer as the temperature began falling. The dilemma of recording low *f*_H_ values with data loggers has been documented previously and is a trade-off between memory, battery life, and the maximum usable tag size for a given fish (Brijs et al., 2019; Zrini and Gamperl, 2021). While a longer recording interval (e.g., 15 s at 100 Hz) is now available with the Star-Oddi's “long ECG” function and helped to combat this issue in the study, caution is warranted when working with fish with low intrinsic *f*_H_s and at low temperatures.

#### Time Required for Recovery

The implantation of tags requires that procedures such as netting, handling, and anesthesia be used and that the fish undergo surgery, which are all known to be stressful on the fish being tagged (Altimiras and Larsen, 2000; Grans et al., 2014; Raby et al., 2015). A number of studies have provided data on how long it takes for fish to completely recover from the implantation of DSTs. Brijs et al. (2019) and Føre et al. (2021) estimated that it can take as little as 4–6 days for *f*_H_ to reach baseline/steady state values post-surgery/tag implantation. However, based on the present *f*_H_ data, and the long-term data sets provided by Zrini and Gamperl (2021) and Hvas et al. (2020), it is clear that *f*_H_ is often not stable for at least 2 weeks post-surgery/implantation. This conclusion agrees with the HRV data collected in this study. HRV was significantly elevated during the night-time (by up to 2-fold) for the first 17 days post-surgery/implantation (Figure 4D).

#### Factors Affecting Heart Rate and HRV

After 2 weeks of recovery, mean *f*_H_ at 12°C was ~ 47 bpm, and this parameter was clearly dependent on water temperature and time of day over the course of this study. For example, the pattern of changes in mean *f*_H_ and temperature was very similar (Figures 4B,C); *f*_H_ during both the day-time and night-time was significantly related to temperature (*p* < 0.001) and had Q_10_ values from 9 to 19°C of ~2.0 and 2.6, respectively (Figure 5A); and *f*_H_ was ~ 12 bpm higher in the day-time as compared to the night-time (Figure 5A). The *f*_Hs_ reported for our salmon at various temperatures are extremely similar to those recorded by several authors for free-swimming rainbow trout (*O. mykiss*) and salmon fitted with data loggers once temperature is taken into account (temperatures 4–14°C; Brijs et al., 2018, 2019; Hjelmstedt et al., 2020; Hvas et al., 2020, 2021; Føre et al., 2021; Svendsen et al., 2021). However, they are much lower than those measured in salmon of similar stocks fitted with blood flow probes in respirometers after 1–2 days of recovery (e.g., see Penney et al., 2014). These latter values are ~15 bpm higher than measured in the sea-cages, and thus, this cautions against using data from such lab-based experiments to estimate the *f*_H_ of fish in sea-cages. That there was a diurnal difference in *f*_H_ in the present study is also consistent with the literature (see Aissaoui et al., 2000 and references herein). Data from previous studies appear to suggest that the difference in *f*_H_ in free-swimming salmon between the day-time and night-time increases with water temperature; i.e., it was reported to be ~5 bpm at 4–5°C (Føre et al., 2021; Svendsen et al., 2021), 10 bpm at 10°C (Hjelmstedt et al., 2020; Hvas et al., 2021), and 25 bpm at 14–15°C (Brijs et al., 2018). However, our data, where the difference in *f*_H_ was consistently ~ 12 bpm in the salmon from ~ 8–19.5°C, does not support this conclusion. Brijs et al. (2018) suggested, based on other studies, that the primary factor determining this circadian difference in *f*_H_ was diurnal changes in activity. However, it is clear that this was not the case in this study where both *f*_H_ and acceleration (swimming activity/swimming speed) were measured simultaneously. External acceleration did not differ between night and day (Figure 5C), and the *R*^2^-values on the relationships between *f*_H_ and EA were < 0.1 (Figure 5D). Thus, the data suggest that mechanisms directly related to the circadian rhythm of the fish, rather than exercise-induced changes in metabolism, were responsible for these diurnal differences. This conclusion is consistent with that of Aissaoui et al. (2000), who indicated that diurnal variations in *f*_H_ in fishes are predominantly due to an endogenous circadian rhythm based primarily on external light reception. At present, the mechanism(s) mediating this effect in fish is/are not completely understood. However, Marchant and Farrell (2019) recently showed that the pacemaker cells of the heart have both membrane and calcium “clocks,” and suggest that the latter is involved in the control of heart rate with changes in temperature.

Although there was a very weak overall relationship between EA (activity) and *f*_H_ (Figure 5D), it was clear between August 25th and September 9th that *f*_H_, EA and the percentage of time spent non-steady swimming were all elevated during the day. Again, this may be related to the increased swimming activity of the fish when they were not being fed, as *f*_H_ increases in a linear fashion with swimming speed (e.g., refer to Zrini and Gamperl, 2021) to meet increased metabolic demands of this fish.

Heart rate variability was high during the first 2 weeks post-surgery and increased during the day and the night between August 30th and when the salmon were harvested. This latter increase was clearly related to the decrease in water temperatures over this period as HRV was negatively correlated at both times of day with water temperature (Figure 5B). This result is difficult to explain given recent publications showing that HRV consists of oscillatory components caused by periodic vagal inhibition of the heart beat (Campbell and Egginton, 2007) and that cholinergic tone changes little, or increases only slightly, in rainbow trout with temperature (at least until temperatures approach the critical thermal maximum for the fish; CT_Max_) (Ekström et al., 2014; Gilbert et al., 2019). It is possible that these disparate results are related to the recordings being made on fully recovered free-swimming fish vs. fish in a swim tunnel shortly after surgery, but doubtful. Clearly, long-term ECG data need to be recorded under different temperatures and other conditions, and the results of HRV (as calculated in this study) and power spectral analyses (Campbell et al., 2006; Campbell and Egginton, 2007) compared, before the HRV data that can be generated from the ECG data can be appropriately interpreted with regards to the biology/culture of free-swimming fish. For example, why was HRV higher during the night than during the day given that there was no difference in EA/activity (Figure 5B)?

### What Does This “Real World Experiment” Tell us?

The data reported in this experiment were recorded during a heat wave in Newfoundland where surface water temperatures in the sea-cages exceeded 18°C for approximately one-half of the days in August, oxygen levels in some of the cages on the south coast of Newfoundland approached 70% of air saturation, and significant (up to 100%) mortalities were reported at some cage-sites. A report commissioned to determine the cause of the mortalities (Burke et al., 2020) concluded that these environmental conditions were the primary cause of the large number of salmon deaths. However, more recent data on the effects of these conditions on fish physiology (Gamperl et al., 2020, Zanuzzo et al., 2020; Beemelmanns et al., 2021a,b,c), and contained in this study, do not support this assertion. For example, the salmon in the cage in which we had fish did not experience temperatures > 19.5°C; large (> 800 g) salmon from similar stocks have been shown to tolerate elevated temperatures as high as 21°C for prolonged periods when combined with water O_2_ levels of 60–70% saturation (lower than the lowest O_2_ levels reported in the cages) without mortalities (Gamperl et al., 2020); salmon from Norway and Tasmania can tolerate prolonged exposure to similar temperatures (Hvas et al., 2017; Tromp et al., 2018); the critical oxygen tension/limiting oxygen saturation (LOS) for Atlantic salmon is ~63–67% at 22°C (Barnes et al., 2011; Remen et al., 2013), well below the water oxygen level reported in the cages by Burke et al. (2020); and all the salmon implanted with data loggers that survived for several weeks post-implantation experienced similar conditions to those reported in the summer of 2019. Thus, there were likely other complicating factors. While the Burke et al. (2020) report does suggest that algal blooms, sea lice treatments (and possibly even infectious salmon anemia, ISA) may have contributed to the mortalities, the former is unlikely given the minimum water air saturation levels that were reported (> 70%). However, the salmon at most sites were being treated for sea lice (Burke et al., 2020), and this may have been a critical/key factor. Overton et al. (2018) reported that sea lice treatments at cooler temperatures can result in salmon mortalities ranging from 15 to 30%. A recent study showed that “high” levels of lice infestation (6.8 ± 0.4 lice per fish) decreased the survival probability of post-smolt salmon at 19°C by 25% (Godwin et al., 2020). In fact, the interpretation that cage-site losses of salmon at high temperatures may result from temperature × pathogen interactions is supported by recent data for other fishes. For example, although significant mortalities occurred in southern Newfoundland when Atlantic cod cage-sites got to 19°C at the surface (16–17°C at 5 m) in 2003 (Gollock et al., 2006), Zanuzzo et al. (2019) showed that the critical thermal maximum (CT_max_) and incremental temperature maximum (IT_max_) of Atlantic cod are much greater than these values (22.5 and 21.7°C, respectively), and that IT_max_ for this species was not impacted by moderate hypoxia (70% air saturation). A relationship between increasing water temperature and amoebic gill disease (AGD) prevalence has been noted in Atlantic salmon cultured in several countries, and Benedicenti et al. (2019) showed that salmon reared at higher temperatures (10 vs. 15°C) had earlier infections and more severe parasite loads. Finally, Genin et al. (2020) suggest that while heat waves (“warming events”) may not directly kill coral reef fish, bacterial infections that occur following these events can result in mass mortality events.

### Summary and Perspectives

We implanted large (~2.5 kg) salmon with data loggers/data storage tags (DSTs) that recorded depth, temperature, ECGs (heart rate), and 3-D acceleration (activity) during the summer of 2019 to better understand how conditions during this time of year affect their distribution, behavior, and physiology in sea-cages. This study clearly shows the usefulness of DSTs that provide information on several parameters simultaneously (such as those developed by Star-Oddi and the most recent tag developed through an Australian collaboration; refer to Shen et al., 2020) for studying aspects of salmon (fish) biology and welfare in culture. Specifically, this study has provided invaluable information on how various factors (photoperiod, temperature, and starvation/food restriction) affect Atlantic salmon in sea-cages. Further, these tags are continuously undergoing development to improve the data they collect and their usefulness for studies on the biology of wild and culture fishes. For example, Star-Oddi: has recently increased the maximum recording length for ECGs to 18 s; is now producing tags with the capability to record acceleration at 10 vs. 1 Hz [which will allow for the monitoring of a wider array of fish behaviors (Brown et al., 2013)]; and has developed a method to correct for changes in acceleration values if the tag shifts/rotates during long-term studies (as occurred with DST MAL0011 in the present study).

Our data, in combination with other recently published data (Gamperl et al., 2020; Zanuzzo et al., 2020; Beemelmanns et al., 2021a,b,c; and see references above), also provide vital/key new information on how conditions at salmon cage-sites during the summer/early fall are likely to impact these fish in Atlantic Canada and suggest that a multi-faceted approach may/will be needed to prevent potential losses at salmon cage-sites under current climate change scenarios (see IPCC, 2019). Given recent data which suggest that Atlantic salmon of Saint John River stock can survive long-term exposure to temperatures of > 21°C in combination with moderate hypoxia (60–70% air saturation) and that unlike fish in Tasmania (Stehfest et al., 2017; Wade et al., 2019) have not yet experienced these temperatures, it is clear that selective breeding for upper thermal tolerance (given its effects on food consumption, growth, and stress biomarkers; Gamperl et al., 2020; Beemelmanns et al., 2021a,b) is not only needed to improve production in the summer and to prevent losses that are predicted to get worse given rising average ocean temperatures and more frequent/severe heat waves. Better vaccines against bacterial and viral diseases and mitigating strategies/treatments for pathogen outbreaks (incl. sea lice) that cause minimal stress must also be developed. This is particularly true since recent studies show that the humoral and cellular immune response of the Atlantic salmon does not appear to be impacted by long-term exposure to high temperatures (Zanuzzo et al., 2020); i.e., genetic improvements to enhance these components of the immune response may only have limited effect.

## Data Availability Statement

The raw data supporting the conclusions of this article will be made available by the authors, without undue reservation.

## Ethics Statement

All experimental procedures were performed in compliance with the guidelines of the Canadian Council on Animal Care and were approved by the Memorial University of Newfoundland's Animal Care Committee. Written informed consent was obtained from the owners for the participation of their animals in this study.

## Author Contributions

AG conceived the project, secured funding, and wrote the first draft of the paper. AG, ZZ, and RS designed the experiment. ZZ and RS conducted the experiments, analyzed the data, and revised the manuscript. All authors interpreted the data and approved the submission of this article.

## Conflict of Interest

The authors declare that the research was conducted in the absence of any commercial or financial relationships that could be construed as a potential conflict of interest.

## Publisher's Note

All claims expressed in this article are solely those of the authors and do not necessarily represent those of their affiliated organizations, or those of the publisher, the editors and the reviewers. Any product that may be evaluated in this article, or claim that may be made by its manufacturer, is not guaranteed or endorsed by the publisher.

## Supplementary Material

Supplementary Figure 1 Individual depth and temperature profiles for fish 1 **(A)** −7 **(F)** created by recording these parameters every 5 min (288 times per day).

Supplementary Figure 2 Individual day-time (open circles, dotted lines) and night-time (filled circles, solid lines) relationships between heart rate, heart rate variability, external acceleration, and temperature of individual fish (1: black; 2: red; 3: green; 4: blue; and 5: purple).
