# Supplementary figures and images for: Atlantic Salmon (Salmo salar) Cage-Site Distribution, Behavior, and Physiology During a Newfoundland Heat Wave
Source: Front Physiol. 2021 Aug 24;12:719594. doi: 10.3389/fphys.2021.719594 (PMC8421689; doi:10.3389/fphys.2021.719594)

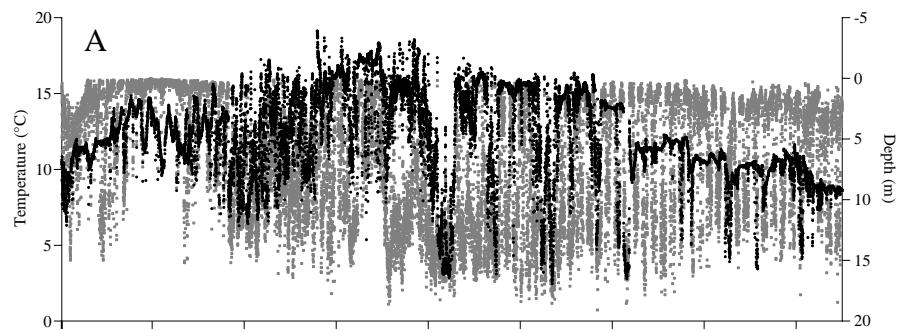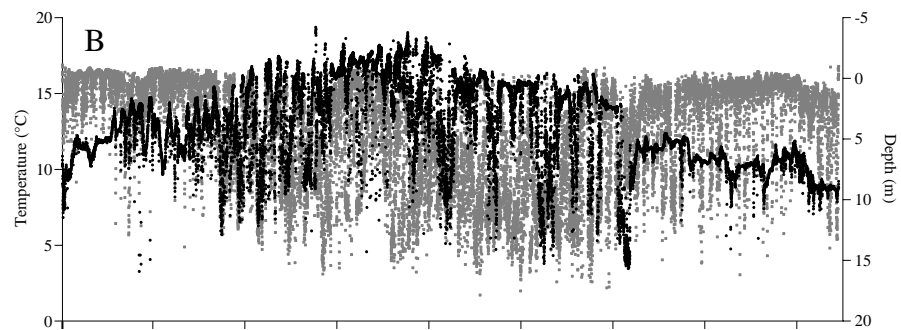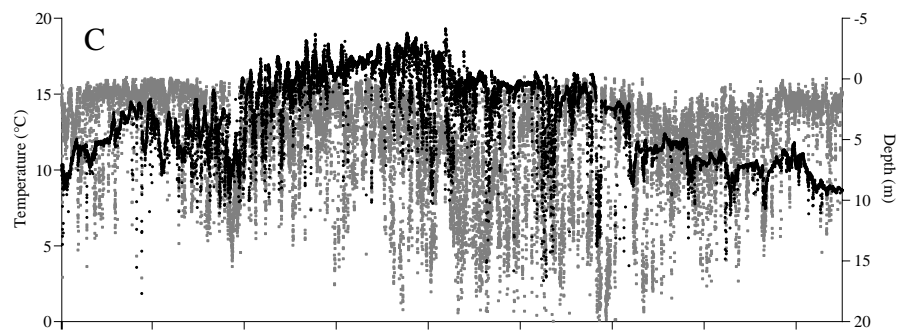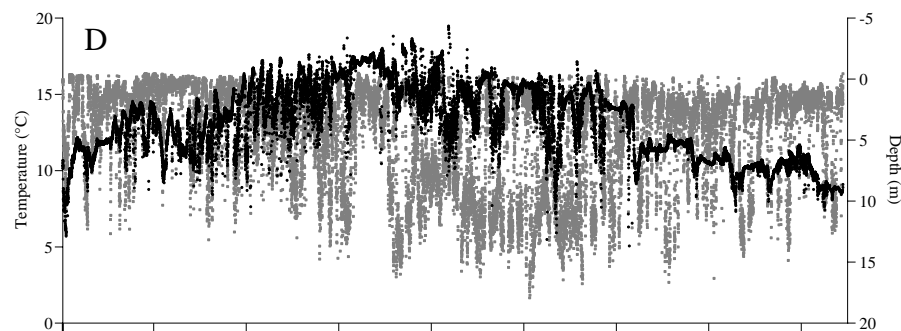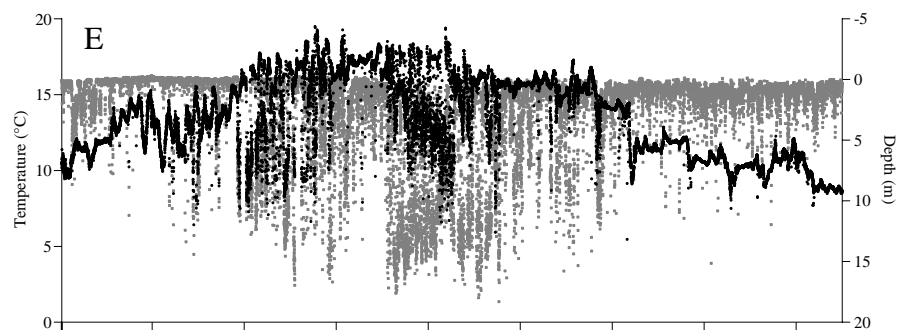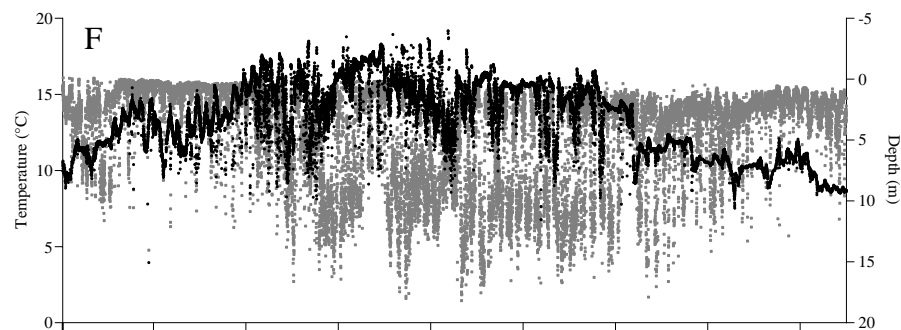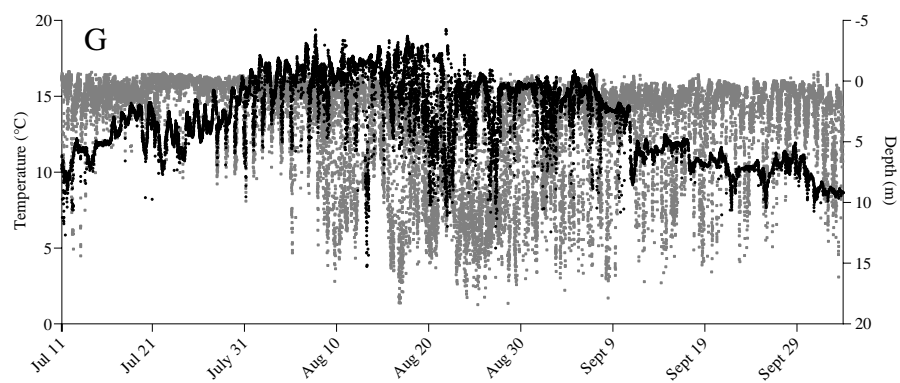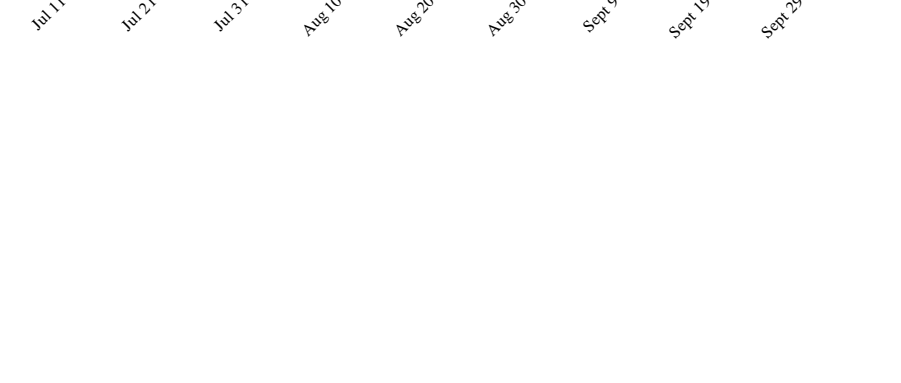

Supplement: Supplementary Figure 1 — Individual depth and temperature profiles for fish 1 (A) −7 (F) created by recording these parameters every 5 min (288 times per day). [file Data_Sheet_1.PDF]

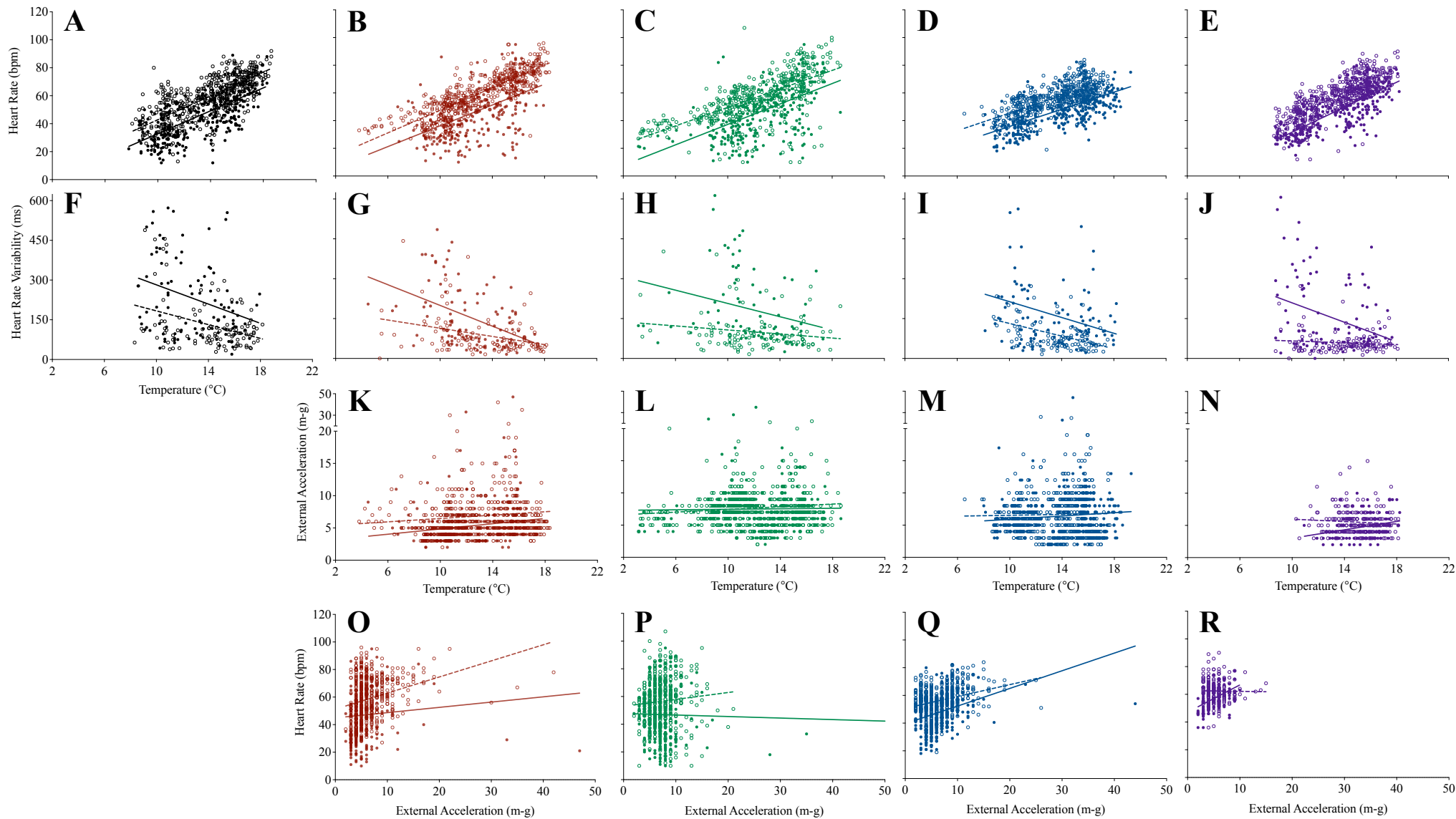

Supplement: Supplementary Figure 2 — Individual day-time (open circles, dotted lines) and night-time (filled circles, solid lines) relationships between heart rate, heart rate variability, external acceleration, and temperature of individual fish (1: black; 2: red; 3: green; 4: blue; and 5: purple). [file Data_Sheet_2.PDF]
