# Supplementary material for: Atlantic Salmon (Salmo salar) Cage-Site Distribution, Behavior, and Physiology During a Newfoundland Heat Wave
Source: Front Physiol. 2021 Aug 24;12:719594. doi: 10.3389/fphys.2021.719594 (PMC8421689; doi:10.3389/fphys.2021.719594)
Supplement: Supplementary file 3 [file Data_Sheet_3.PDF]

Concerns about climate change (rising ocean temperatures, increases in the frequency and severity of heat waves and hypoxic events) are being realized in many regions, and having impacts on wild and cultured marine fishes. Given the valuable data that has recently been collected on conditions and fish welfare at trout and salmon sea-cage aquaculture sites using data storage tags (DSTs), we implanted Atlantic salmon at a cage-site on the south coast of Newfoundland (Canada) with Star-Oddi DSTs in the early summer of 2019 that recorded the fish's temperature, depth, swimming activity and heart rate. These are particularly valuable data as this research coincided with a marine heat wave event in this region. This research shows that although the fish typically selected temperatures from 14-18°C and moved deeper as surface temperatures warmed, they continued to use the full range of depths available during the warmest part of the summer. Further, it shows that salmon physiology and behavior were influenced by cage-site operations and environmental conditions. The data provide: additional evidence that DSTs are an effective tool for understanding the physiology, behavior and welfare of free-swimming fishes; and key insights into the biggest challenges to salmon welfare during such events.
